# Contribution of community health workers to primary health care performance in Brazil

**DOI:** 10.11606/s1518-8787.2020054002327

**Published:** 2020-11-27

**Authors:** Alaneir de Fátima dos Santos, Hugo André da Rocha, Ângela Maria de Lourdes Dayrell de Lima, Daisy Maria Xavier de Abreu, Érica Araújo Silva, Lucas Henrique Lobato de Araújo, Isabela Cristina Carreiro Cavalcante, Antônio Thomaz Gonzaga da Matta-Machado

**Affiliations:** I Universidade Federal de Minas Gerais Faculdade de Medicina Departamento de Medicina Preventiva e Social Belo HorizonteMG Brasil Universidade Federal de Minas Gerais. Faculdade de Medicina. Departamento de Medicina Preventiva e Social. Belo Horizonte, MG, Brasil; II Universidade Federal de Minas Gerais Faculdade de Medicina Programa de Pós-Graduação em Saúde Pública Belo HorizonteMG Brasil Universidade Federal de Minas Gerais. Faculdade de Medicina. Programa de Pós-Graduação em Saúde Pública. Belo Horizonte, MG, Brasil; III Universidade Federal de Minas Gerais Faculdade de Medicina Núcleo de Educação em Saúde Coletiva Belo HorizonteMG Brasil Universidade Federal de Minas Gerais. Faculdade de Medicina. Núcleo de Educação em Saúde Coletiva. Belo Horizonte, MG, Brasil

**Keywords:** Primary Health Care, Community Health Workers, Health Services Research, Brazil

## Abstract

**OBJECTIVE:**

To associate the strength of community health workers interventions with primary health care strategies for women’s and children’s health, diabetes, and hypertension.

**METHODS:**

This is a cross-sectional study assessing 29,778 family health teams working in primary health care in Brazil in 2014. The association between community health workers activity levels and primary health care facilities was analyzed using multiple logistic regression.

**RESULTS:**

We found higher levels of community health workers activities strongly associated with primary health care practices (OR = 6.88) for those activities targeting hypertension management, followed by children’s health (OR = 6.56), and women’s health (OR = 6.21).

**CONCLUSIONS:**

At a time when Brazil discusses whether community health workers should or should not remain in the same scale-up and skill level as they currently are, our results reinforce the importance of these workers for the care model advocated by the Brazilian Unified Health System.

## INTRODUCTION

Community health workers (CHW) have had an essential role in bridging between the communities within which they act and primary health care services. CHW are commonly defined as health professionals who deliver care services out of health facilities and are trained in some way in the context of intervention strategies^1^. Given their importance as trusted members of the communities they serve, responding to local societal and cultural norms and customs to ensure community acceptance and ownership, the number of CHW has grown over time^2^. By 2014, the number of CHW was estimated at approximately 5 million worldwide, among which 2.3 million were located in India, and from 85.000 to 200.00 in the United States^3^.

The literature presents strong evidences of these professionals positive contribution to different healthcare aspects, such as: promoting immunization, detecting and improving the outcomes of acute respiratory diseases and malaria, and reducing child morbidity and mortality^1,2,4,5^.

In Brazil, the CHW plays a pivotal role in the family health team (FHT) together with one physician, one nurse, and nurse technicians. In 2017, 257,872 CWH were working in primary health care serving 126,334,402 people – 61.29% of the Brazilian population. The articulation of CHW and FHT has been successful in the country, achieving outstanding results^6^.

Despite evidences of CHW relevance at international level, their essential role within FHT is an ongoing discussion in Brazil – particularly by health services managers, concerned with their high number and cost, or even their permanence within the country primary care structure^7^. A recent review addressing CHW function in Brazil urge the need for them to develop activities for health promotion, disease prevention (especially those most prevalent in the region), and health surveillance through home visits and educational actions, including epidemiological investigation of suspected cases. It also indicates that CHW must conduct home visits at the frequency established in the team planning and based on the population health needs to monitor families’ and individuals’ situation, particularly those with diseases and conditions requiring more home visits^8^.

This article aims to contribute to this discussion by analyzing the association of CHW household activities and FHT practices targeting women’s and children’s health, diabetes, and hypertension. For that, we will rely on data from the *Programa Nacional de Melhoria do Acesso e da Qualidade da Atenção Básica* (National Program for Access and Quality Improvement in Primary Care – PMAQ-AB), cycle II (2014), which contains data regarding care practices developed by the FHT.

## METHODS

This is a cross-sectional study employing PMAQ-AB cycle II external evaluation database, organized by the Ministry of Health. By continuous and progressive processes, PMAQ-AB aims to expand the capacity of the three spheres of government to provide services while ensuring a quality standard comparable at national, regional, and local levels^9^. Considering PMAQ-AB scope, interviews were conducted with 29,778 FHT professionals previously chosen by the teams and distributed throughout Brazilian states, who joined the program in 2014.

### Variables selection

Variables of the PMAQ-AB cycle II questionnaire were used to evaluate practices developed by the family health team (FHT) targeting four care strategies: women’s health, children’s health, diabetes care, and hypertension care.

In Brazil, several professionals conduct home visits. As observed in Table 1, doctors, nurses, mid-level professionals, other professionals, and community health agents conducted visits in 2013 and 2014, among which 92.3%, on average, were performed by community health workers (CHW). Considering that, we assumed that home-based activities are primarily performed by CHW.

**Table 1:** Distribution of number of home visits by type of professional, 2013 and 2014, Brazil.

Type of professional	2013		2014	
	
	n	%	n	%
CHW	343,023,069	92.3	318,000,654	92.4
Doctors	4,549,350	1.2	4,139,077	1.2
Nurses	7,441,938	2.0	6,658,291	1.9
Other undergraduate-degree professionals	1,974,242	0.5	2,115,596	0.6
Mid-level professionals	14,825,448	4.0	13,413,350	3.9

Total	371,814,047	100.0	344,326,968	100.0

CHW: community health workers.

Source: Department of Primary Care, Ministry of Health, Brazil.

To represent the scope of activities conducted by FHT at patients’ homes, we created an independent variable called *scope of home activities* that allow us to estimate CHW’s activities, as they are the main responsible for home visits and active search. For that, the following activities were considered: active search for Pap smear test delay, breast cancer screening, prenatal testing, women in up to 10 days postpartum, children under 2 years of age, preterm and low-birth weight infants, delayed vaccination, delayed child’s development, non-treated tuberculosis cases; surveillance of new intra-household tuberculosis contacts, untreated and new cases of Hansen’s disease, mental health, alcohol and drug abuse, hypertension, and diabetes.

Activities related to visits frequency, considering risk and vulnerability assessment, visits scheduling according to team’s priorities, and population education on the adequate use and quality of water reservoirs were also included.

The *scope of home activities* variable was calculated by the sum of FHT activities conducted at patients’ homes, following PMAQ-AB categorization into low activity (from 0 to 49%), medium activity (50 to 69%), and high activity (above 70%), according to frequency distribution. Both absolute and relative frequency, as well as frequency distribution according to Brazilian federal units and regions were used to analyze this variable.

The dependent variables used in this study are related to four health care strategies – women’s and children’s health, hypertension, and *diabetes mellitus*– and practices targeting these four strategies. These variables were assigned reference category of low level of practices and distributed by geographic regions.

### Data Analysis

Data analysis included descriptive and inferential statistics. The association between the scope of home activities conducted by the FHT and primary health practices was analyzed by multiple logistic regression considering different Brazilian regions and health care strategies. The evaluation was initially based on all variables proposed.

All analyses adopted a *p-*value < 0.05 for statistical significance and a 95% confidence interval (CI), calculating the respective odds ratio (OR). Each variable estimated OR and 95% CI were adjusted for the effect of other variables studied in the model. Hosmer-Lemeshow goodness-of-fit tests were applied to final multiple model. All statistical analyses were performed in the Statistical Package for the Social Sciences- SPSS version 15.0. The project was approved by the Research Ethics Committee of UFMG under opinion no. 1.275.911.

## RESULTS

We analyzed the data from 29,788 family health team (FHT) professionals. Table 2 shows that 48.5% of FHT community health workers (CHW) had high activity level, with great variation within the country. The Midwest (55.9%) and Northeast regions (50.6%) showed high levels of CHW activity (Table 2). In turn, the Midwest region showed the lowest frequency of lower levels of CHW activity (19.5%) whereas the Southeast region showed the highest frequency (30.1%).

**Table 2:** Distribution of CHW’s level of activity by regions. PMAQ, 2014, Brazil.

Regions	Level of activity					

	Low		Medium		High	
		
	n	%	n	%	n	%
North	564	26.1	589	27.3	1,007	46.6
Northeast	2,813	26.1	2,510	23.3	5,445	50.6
Southeast	3,036	30.1	2,359	23.4	4,705	46.6
South	1,216	27.0	1,252	27.8	2,041	45.3
Midwest	437	19.5	551	24.6	1,253	55.9

Total	8,066	27.1	7,261	24.4	14,451	48.5

CHW: community health workers.

PMAQ: *Programa Nacional de Melhoria do Acesso e da Qualidade da Atenção Básica.*

Primary health practices also presented different results, as shown in Table 3. FHT reached a medium-high level in 52.8% of practices targeting women’s health; for children’s health this level was 63.3%, for hypertension 71.1%, and for diabetes 79.9%. As for regions, the Southeast presented the best performance for practices targeting all health care strategies – *diabetes mellitus* (83.5%), hypertension (75.3%), women’s health (59.6%), and children’s health (69.8%). The North region presented the worst performance for women’s (67.1%) and children’s health (59.2%), but reached medium-high levels for hypertension (57.8%) and *diabetes mellitus* (69.5%).

**Table 3:** Distribution of level of practice by healthcare strategies and regions. PMAQ, 2014, Brazil.

Regions	Healthcare strategies	Level of Practice			

		Low		Medium-high	
	
		n	%	n	%
North	HTN	912	42.2	1,248	57.8
	DM	659	30.5	1,501	69.5
	Women	1,449	67.1	711	32.9
	Children	1,279	59.2	881	40.8

Northeast	HTN	2,820	26.2	7,948	73.8
	DM	1,887	17.5	8,881	82.5
	Women	5,247	48.7	5,521	51.3
	Children	4,050	37.6	6,718	62.4

Southeast	HTN	2,492	24.7	7,608	75.3
	DM	1,662	16.5	8,438	83.5
	Women	4,079	40.4	6,021	59.6
	Children	3,053	30.2	7,047	69.8

South	HTN	1,562	34.6	2,947	65.4
	DM	1,171	26.0	3,338	74.0
	Women	2,033	45.1	2,476	54.9
	Children	1,501	33.3	3,008	66.7

Midwest	HTN	818	36.5	1,423	63.5
	DM	607	27.1	1,634	72.9
	Women	1,256	56.0	985	44.0
	Children	1,053	47.0	1,188	53.0

Total	HTN	8,604	28.9	21,174	71.1
	DM	5,986	20.1	23,792	79.9
	Women	14,064	47.2	15,714	52.8
	Children	10,936	36.7	18,842	63.3

HTN: hypertension; DM: *diabetes mellitus*.

Table 4 shows the association, using logistic regression, between activities focusing on primary care practices and CHW activities. We found a positive association between all analyzed strategies (hypertension, *diabetes mellitus*, and women’s and children’s health) and the level of CHW activity. Higher levels of CHW activity were associated with better health practices for the corresponding strategy. The strongest association between high CHW activity and the health practice occurred in activities targeting hypertension management (OR = 6.88), followed by children’s health (OR = 6.56), women’s health (OR = 6.21), and diabetes (OR = 5.64). Even medium levels of CHW activity showed better health practices in the different strategies, with OR ranging from 2.75 to 2.11.

**Table 4:** Logistic regression of health strategies and CHW’s level of activity. Brazil, 2014.

Strategy	Level	B	p	OR	95%CI
HTN	Low			1	-
	Medium	1.01	0.001	2.75	2.52–3.0
	High	1.92	0.001	6.88	6.13–7.71

DM	Low			1	-
	Medium	0.77	0.001	2.16	2.01–2.32
	High	1.73	0.001	5.64	5.23–6.07

Women’s health	Low			1	-
	Medium	0.74	0.001	2.11	1.97–2.26
	High	1.82	0.001	6.21	5.84–6.59

Children’s health	Low			1	-
	Medium	0.78	0.001	2.19	2.05–2.33
	High	1.88	0.001	6.56	6.12–7.04

CHW: community health workers HTN: hypertension; DM: *diabetes mellitus*.

* Reference category: low levels of activity; response category: medium-high levels of activity.

Table 5 shows associations for the different strategies according to regions. The Southeastern region showed the highest association rates for all health practices analyzed – women’s health (OR = 10.12), *diabetes mellitus* (OR = 8.75), hypertension (OR = 8.09), and children’s health (OR = 7.10). The other regions also showed strong associations, exceeding 4 in most health practices.

**Table 5:** Logistic regression of health practices and CHW’s activity levels by region. Brazil, 2014.

Strategy	Regions	Level	B	p	OR	95%CI
HTN	North	Low			1.00	-
		Medium	0.95	0.00	2.59	2.04–3.30
		High	2.00	0.00	7.41	5.88–9.33

	Northeast	Low			1.00	-
		Medium	0.67	0.00	1.95	1.74–2.19
		High	1.52	0.00	4.57	4.11–5.08

	Southeast	Low			1.00	-
		Medium	0.84	0.00	2.31	2.06–2.59
		High	2.09	0.00	8.09	7.17–9.13

	South	Low			1.00	-
		Medium	0.52	0.00	1.69	1.44–1.98
		High	1.58	0.00	4.87	4.16–5.69

	Midwest	Low			1.00	-
		Medium	0.82	0.00	2.27	1.75–2.95
		High	1.96	0.00	7.12	5.60–9.04

DM	North	Low			1,00	-
		Medium	0.77	0.00	2,16	1.70–2.73
		High	1.96	0.00	7,11	5.58–9.05

	Northeast	Low			1,00	-
		Medium	0.68	0.00	1,98	1.75–2.25
		High	1.47	0.00	4,36	3.87–4.92

	Southeast	Low			1.00	-
		Medium	0.89	0.00	2.43	2.13–2.77
		High	2.17	0.00	8.75	7.55–10.14

	South	Low			1.00	-
		Medium	0.61	0.00	1.84	1.56–2.17
		High	1.74	0.00	5,67	4.77–6.75

	Midwest	Low			1.00	-
		Medium	0.77	0.00	2.15	1.66–2.79
		High	1.74	0.00	5.71	4.48–7.26

Women	North	Low			1.00	-
		Medium	0.54	0.00	1.71	1.24–2.36
		High	2.04	0.00	7.72	5.84–10.20

	Northeast	Low			1.00	-
		Medium	0.64	0.00	1.90	1.70–2.13
		High	1.61	0.00	4.99	4.52–5.51

	Southeast	Low			1.00	-
		Medium	1.02	0.00	2.78	2..49–3.11
		High	2.31	0.00	10.12	9.10–11.26

	South	Low			1.00	-
		Medium	0.76	0.00	2.14	1.82–2.52
		High	1.82	0.00	6.17	5.28–7.22

	Midwest	Low			1.00	-
		Medium	0.59	0.00	1.80	1.34–2.42
		High	1.69	0.00	5.45	4.20–7.06

Children	North	Low			1	-
		Medium	0.51	0.00	1.66	1.27–2.17
		High	1.59	0.00	4.89	3.86–6.20

	Northeast	Low			1	-
		Medium	0.20	0.00	1.22	1.10–1.36
		High	1.14	0.00	3.12	2.83–3.43

	Southeast	Low			1	-
		Medium	0.76	0.00	2.13	1.91–2.38
		High	1.96	0.00	7.10	6.36–7.92

	South	Low			1	-
		Medium	0.47	0.00	1.59	1.36–1.87
		High	1.34	0.82	3.83	3.28–4.48

	Midwest	Low			1	-
		Medium	0.56	0.00	1.74	1.33–2.28
		High	1.67	0.00	5.33	4.19–6.77

HTN: hypertension; DM: *diabetes mellitus*.

* Reference category: low levels of activity; response category: medium-high levels of activity.

## DISCUSSION

This study approached the role of community health workers (CHW) in the Brazilian public health system and their association with health practices. Our most relevant finding was the medium-high level correlation of CHW activity. High CHW activity was positively correlated to health practices in all fields investigated – hypertension, *diabetes mellitus*, children’s health, and women’s health, – indicating that high CHW activity in a given field is associated with better heath.

A systematic review identified that factors related to the nature of tasks and time required, such as human resources management, quality assurance, links with the community, links with the healthcare system, resources, and logistics, influence CHW performance. Good performance was associated to intervention projects involving a combination of incentives, constant supervision, ongoing training, community involvement, and strong coordination and communication between CHW and healthcare professionals^10^. Several studies suggested that being trained and supervised over time, recognized by the community they serve, and working together with other professionals interfered in these workers’ performance^2,11^. Considering that, Brazil still has a long path ahead so that all FHT can entail a high level of CHW activity.

Our findings corroborate those reported by international studies that carefully investigated the correlation between CHW and the outcomes of health care strategies. Previous systematic reviews found that CHW activities effectively promote a wide range of healthy behaviors, such as breast cancer screening and diabetes, hypertension, and asthma self-management, and adherence to drug treatment in patients with HIV/AIDS^12^.

We also found that medium or high levels of CHW activity are associated with improved care practices for hypertension and diabetes. An international review on CHW performance in diabetes and hypertension management in low-income countries found that CHW interventions may improve glycemic control or reduce the risk of disease progression^13^. As for cardiovascular risk, the CHW intervention group showed significant improvements in the lipid profile (total cholesterol, low-density lipoprotein, high-density lipoprotein or triglycerides, blood pressure, glycated hemoglobin) and lower overall risk of cardiovascular diseases than the control group^14^.

A review on CHW role in improving diabetes outcomes conducted in the United States concluded that studies usually support CHW function in promoting better community care for diabetes patients and families, with evidences of significant lower glycated hemoglobin levels^15^. Other studies stressed the emerging evidence of improved glycemic control or reduced risk of disease in community-based participatory research (CBPR) involving CHW, indicating that glycated hemoglobin reduction was greater with higher mean values of CHW activities^16^.

Our study found that medium-high levels of CHW activity improved health practices targeting women’s health. A systematic review on CHW-led women’s groups practicing participatory learning and action in Bangladesh, India, Malawi, and Nepal reported a 37% reduction in maternal mortality^17^. A recent review on CHW efficacy in providing family planning services concluded that they can safely provide contraceptive pills and condoms, emergency contraception, and injectable contraceptives, as well as effectively promoting the Standard Days Method and the Lactational Amenorrhea Method^18^. In Africa, recent studies have reported similar evidence in injectable contraceptives^19^. Community-based distribution programs conducted in Afghanistan increased the use of family planning by three- or four-fold (5-10% to 20-40%) in areas where initial coverage was very low^20^.

A review of 15 studies conducted in the United States found CHW to be effective in increasing adherence to Pap smear and mammography screening when compared to usual care, but not for other types of cancers^21,22^. A recent systematic review evaluating CHW interventions for improving breast cancer screening rates revealed that CHW were effective in certain contexts and populations, especially in urban settings and among subjects of the same ethnicity^23,24^.

Regarding the children’s health, there are evidences that CHW have also reached positive results in promoting vaccination and reducing acute respiratory diseases, when compared with usual care^25^. In this respect, a Cochrane review conducted in 2010 approaching CHW role in referring infectious diseases among pregnant women and children concluded that CHW effectively promoted vaccination, reducing morbidity and mortality when compared to ordinary care^5^. We also found an important association between practices targeting children’s health and care outcomes.

We found different activity levels of CHW and various outcomes of health practices among Brazilian geographic regions: while Southeast and South regions presented a lower level of CHW activity, the Midwest and Northeast showed higher levels. CHW have played meaningful roles in low-income regions, providing the greatest benefit for the most disadvantaged populations; we found this scenario especially within Northeast region, given its high rates of unmet health care needs. Although the program was not solely oriented towards poor regions, it was intended to improve low-income and vulnerable groups access to health care. In this way, CHW are highly respected within communities, given their stable and enduring presence in a family experience with primary care^26^.

As demonstrated in our study, FHT with greater performance on hypertension, diabetes, and women’s and children’s health are more likely to entail greater levels of CHW activity, indicating that CHW presence within the team’s configuration enlarges teams’ actions scope. CHW activities, particularly those related to active search, enable an easier monitoring of the health conditions of populations under FHT responsibility. Given their access and circulation throughout the territory, CHW enables FHT to act on social sphere besides clinical issues. A study evaluating users’ satisfaction with primary health care (PHC) using data from the PMAQ-AB PHC cycle I found that home visits conducted by the CHW maximize user satisfaction^27^. Health home-based activities are predominantly performed by CHW and may be characterized as actions for health promotion. In fact, home visits influence how users perceive the provided care, so the chance of the user evaluating the care as good or very good increases alongside the number of conducted visits^28^.

In total, 93% of FHT participating in PMAQ-AB cycle I scheduled visits based on the criteria of priority groups^29^. A longitudinal perspective on monitoring patients found positive results in detecting and following-up patients with tuberculosis, with 70.6% of the FHT performing an active search^30^. A study analyzing changes concerning PHC attributes between PMAQ-AB first and second cycles reported improvements regarding first contact access and services comprehensiveness. However, the most sensitive attribute of CHW activity is observed longitudinally, whereby CHW may contribute to a better PHC performance by promoting active search and home visits, as well as by strengthening the FHT-population link^31^.

In a context of inequitable access and health care provision, CHW may verify the family situation, its issues, needs, and wishes, determining a community diagnosis and informing the FHT for developing actions. This study has some limitations: information related to CHW activity were collected based on PMAQ-AB, and the questionnaires are often answered by nurses responsible for supervising the CHW. We investigated potential associations considering general health practices within PMAQ cycle II, with questions addressing the staff in charge of home visits. Yet, this study may contribute to a better understanding on how CHW activity may fit into the Brazilian Unified Health System (SUS) and how it affects care outcomes.

## CONCLUSIONS

This study found a strong correlation between CHW activities and health practices targeting hypertension, diabetes, and women’s and child’s health. Our results corroborate that reported by international studies, which found evidence on CHW achieved outcomes. At a time when Brazil discusses whether CHW should or should not remain in the same number and organizational structure as they currently are, our results reinforce the importance of these workers for the care model advocated by SUS.

## References

[1] 1. Lewin S, Dick J, Pond P, Zwarenstein M, Aja GN, Wyk BE, et al. Lay health workers in primary and community health care. Cochrane Database Syst Rev. 2005;(1):CD004015. 10.1002/14651858.CD004015.pub2 15674924

[2] 2. Lehmann U, Sanders D. Community health workers: what do we know about them? The state of the evidence on programmes, activities, costs and impact on health outcomes of using community health workers. Geneva: World Health Organization; 2007 [cited 2018 Nov 28]. Available from: https://www.who.int/hrh/documents/community_health_workers.pdf

[3] 3. Perry HB, Zulliger R, Rogers MM. Community health workers in low-, middle-, and high-income countries: an overview of their history, recent evolution, and current effectiveness. Annu Rev Public Health. 2014;35:399-421. 10.1146/annurev-publhealth-032013-182354 24387091

[4] 4. Landers S, Levinson M. Mounting evidence of the effectiveness and versatility of community health workers. Am J Public Health. 2016;106(4):591-2. 10.2105/AJPH.2016.303099. PMC4816154 26959254

[5] 5. Lewin S, Munabi-Babigumira S, Glenton C, Daniels K, Bosch-Capblanch X, Wyk BE, et al. Lay health workers in primary and community health care for maternal and child health and the management of infectious diseases. Cochrane Database Syst Rev. 2010;2010(3):CD004015. 10.1002/14651858.CD004015.pub3 PMC6485809 20238326

[6] 6. Ministério da Saúde (BR). SAGE - Sala de Apoio à Gestão Estratégica. Brasília, DF; 2016 [cited 2017 Sept 21]. Available from: http://sage.saude.gov.br/?link=paineis/acs/corpao&flt=false&param=null

[7] 7. Melo EA, Mendonça MHM, Oliveira JR, Andrade GCL. Mudanças na Política Nacional de Atenção Básica: entre retrocessos e desafios. Saude Debate. 2018;42 Nº Espec 1:38-51. 10.1590/0103-11042018s103.

[8] 8. Giugliani C, Harzheim E, Duncan MS, Duncan BB. Effectiveness of community health workers in Brazil: a systematic review. J. Ambul Care Manage. 2011;34(4):326-38. 10.1097/JAC.0b013e31822cbdfd 21914989

[9] 9. Ministério da Saúde (BR). Programa de Melhoria do Acesso e da Qualidade na Atenção Básica. Manual instrutivo para as equipes de Atenção Básica (Saúde da Família, Saúde Bucal e Equipes Parametrizadas) e NASF. Brasília, DF; 2013 {cited 2018 Sept 21]. Available from: http://189.28.128.100/dab/docs/portaldab/publicacoes/manual_instrutivo_PMAQ_AB2013.pdf

[10] 10. Kok MC, Dieleman M, Taegtmeyer M, Broerse JEW, Kane SS, Ormel H, et al. Which intervention design factors influence performance of community health workers in low- and middle-income countries? A systematic review. Health Policy Plan. 2015;30(9):1207-27. 10.1093/heapol/czu126 PMC4597042 25500559

[11] 11. Källander K, Strachan D, Soremekun S, Hill Z, Lingam R, Tibenderana J, et al. Evaluating the effect of innovative motivation and supervision approaches on community health worker performance and retention in Uganda and Mozambique: study protocol for a randomized controlled trial. Trials 2015;16:157. 10.1186/s13063-015-0657-6 PMC4432981 25873093

[12] 12. Kim K, Choi JS, Choi E, Nieman CL, Joo JH, Lin FR, et al. Effects of community-based health worker interventions to improve chronic disease management and care among vulnerable populations: a systematic review. Am J Public Health. 2016;106(4):e3-e28. 10.2105/AJPH.2015.302987 PMC4785041 26890177

[13] 13. Allen JK, Himmelfarb CRD, Szanton SL, Bone L, Hill MN, Levine DM. COACH trial: a randomized controlled trial of nurse practitioner/community health worker cardiovascular disease risk reduction in urban community health centers: rationale and design. Contemp Clin Trials. 2011;32(3):403-11. 10.1016/j.cct.2011.01.001 PMC3070050 21241828

[14] 14. Brownstein JN, Chowdhury FM, Norris SL, Horsley T, Jack Jr L, Zhang X, et al. Effectiveness of community health workers in the care of people with hypertension. Am J Prev Med. 2007;32(5):435-47. 10.1016/j.amepre.2007.01.011 17478270

[15] 15. Hunt CW, Grant JS, Appel SJ. An integrative review of community health advisors in type 2 diabetes. J Community Health. 2011;36(5):883-93. 10.1007/s10900-011-9381-7 21344237

[16] 16. Palmas W, March D, Darakjy S, Findley SE, Teresi J, Carrasquillo O, et al. Community health worker interventions to improve glycemic control in people with diabetes: a systematic review and meta-analysis. J Gen Intern Med. 2015;30(7):1004-12. 10.1007/s11606-015-3247-0 PMC4471021 25735938

[17] 17. Hoke TH, Wheeler SB, Lynd K, Green MS, Razafindravony BH, Rasamihajamanana E, et al. Community-based provision of injectable contraceptives in Madagascar: ‘task shifting’ to expand access to injectable contraceptives. Health Policy Plan. 2012;27(1):52-9. 10.1093/heapol/czr003 21257652

[18] 18. Prost A, Colbourn T, Seward N, Azad K, Coomarasamy A, Copas A, et al. Women’s groups practising participatory learning and action to improve maternal and newborn health in low-resource settings: a systematic review and meta-analysis. Lancet. 2013;381(9879):1736-46. 10.1016/S0140-6736(13)60685-6 PMC3797417 23683640

[19] 19. HIP ~ Family Planning High Impact Practices. Community health workers: bringing family planning services to where people live and work. Baltimore, MD: Johns Hopkins University, HIP Family Planning High Impact Practices; c2020 [cited 2018 Nov 28]. Available from: https://www.fphighimpactpractices.org/briefs/community-health-workers/

[20] 20. Huber D, Saeedi N, Samdi, AK. Achieving success with family planning in rural Afghanistan. Bull World Health Organ. 2010;88(3):227-31. 10.2471/BLT.08.059410 PMC2828781 20428392

[21] 21. Fernández ME, Gonzales A, Tortolero-Luna G, Williams J, Saavedra-Embesi M, Chan W, et al. Effectiveness of Cultivando la Salud: a breast and cervical cancer screening promotion program for low-income Hispanic women. Am J Public Health. 2009;99(5):936-43. 10.2105/AJPH.2008.136713 PMC2667857 19299678

[22] 22. Engelstad LP, Stewart S, Otero-Sabogal R, Leung MS, Davis PI, Pasick RJ. The effectiveness of a community outreach intervention to improve follow-up among underserved women at highest risk for cervical cancer. Prev Med. 2005;41(3-4):741-8. 10.1016/j.ypmed.2005.06.003 16125761

[23] 23. Wells KJ, Luque JS, Miladinovic B, Vargas N, Asvat Y, Roetzheim RG, et al. Do community health worker interventions improve rates of screening mammography in the United States? A Systematic review. Cancer Epidemiol Biomarkers Prev. 2011;20(8):1580-98. 10.1158/1055-9965.EPI-11-0276 PMC3153589 21653645

[24] 24. Crump SR, Shipp MPL, McCray GG, Morris SJ, Okoli JA, Caplan LS, et al. Abnormal mammogram follow-up: do community lay health advocates make a difference? Health Promot Pract. 2008;9(2):140-8. 10.1177/1524839907312806 18340089

[25] 25. Glenton C, Scheel IB, Lewin S, Swingler GH. Can lay health workers increase the uptake of childhood immunisation? Systematic review and typology. Trop Med Int Health. 2011;16(9):1044-53. 10.1111/j.1365-3156.2011.02813.x 21707877

[26] 26. Wadge H, Bhatti Y, Carter A, Harris M, Parston G, Dazi A. Brazil’s Family Health Strategy: using community health care workers to provide Primary Care. New York: The Commonwealth Fund; 2016 [cited 2018 Oct 29]. Available from: https://www.commonwealthfund.org/publications/case-study/2016/dec/brazils-family-health-strategy-using-community-health-care-workers

[27] 27. Protasio APL, Gomes LB, Machado LS, Valença AMG. Satisfação do usuário da Atenção Básica em Saúde por regiões do Brasil: 1º ciclo de avaliação externa do PMAQ-AB. Cienc Saude Coletiva. 2017;22(6):1829-44. 10.1590/1413-81232017226.26472015 28614503

[28] 28. 28 Abreu DMX, Araújo LHL, Reis CMR, Lima AMLD, Santos AF, Jorge AO, et al. Percepção dos usuários sobre o cuidado prestado por equipes participantes do Programa Nacional de Melhoria do Acesso e da Qualidade da Atenção Básica no Brasil. Epidemiol Serv Saude. 2018;27(3):e2017111. 10.5123/S1679-49742018000300002 30183866

[29] 29. Teixeira MB, Casanova A, Oliveira CCM, Ensgtrom EM, Bodstein RCA. Avaliação das práticas de promoção da saúde: um olhar das equipes participantes do Programa Nacional de Melhoria do Acesso e da Qualidade da Atenção Básica. Saude Debate. 2014;38 Nº Espec:52-68. 10.5935/0103-1104.2014S005

[30] 30. Pelissari DM, Bartholomay P, Jacobs MG, Arakaki-Sanchez D, Anjos DSO, Costa MLS, et al. Oferta de serviços pela atenção básica e detecção da incidência de tuberculose no Brasil. Rev Saude Publica. 2018;52:53. 10.11606/s1518-8787.2018052000131 PMC5953548 29791528

[31] 31. Lima JG, Giovanella L, Fausto MCR, Bousquat A, Silva EV. Atributos essenciais da Atenção Primária à Saúde: resultados nacionais do PMAQ-AB. Saude Debate. 2018;42 Nº Espec 1:52-66. 10.1590/0103-11042018s104

